# Is the Sensorimotor Cortex Relevant for Speech Perception and Understanding? An Integrative Review

**DOI:** 10.3389/fnhum.2016.00435

**Published:** 2016-09-21

**Authors:** Malte R. Schomers, Friedemann Pulvermüller

**Affiliations:** ^1^Brain Language Laboratory, Department of Philosophy and Humanities, Freie Universität BerlinBerlin, Germany; ^2^Berlin School of Mind and Brain, Humboldt-Universität zu BerlinBerlin, Germany

**Keywords:** speech perception, sensorimotor integration of speech, motor cortex, somatosensory cortex, articulatory features, multivariate pattern analysis (MVPA), transcranial magnetic stimulation (TMS), embodied cognition

## Abstract

In the neuroscience of language, phonemes are frequently described as multimodal units whose neuronal representations are distributed across perisylvian cortical regions, including auditory and sensorimotor areas. A different position views phonemes primarily as acoustic entities with posterior temporal localization, which are functionally independent from frontoparietal articulatory programs. To address this current controversy, we here discuss experimental results from functional magnetic resonance imaging (fMRI) as well as transcranial magnetic stimulation (TMS) studies. On first glance, a mixed picture emerges, with earlier research documenting neurofunctional distinctions between phonemes in both temporal and frontoparietal sensorimotor systems, but some recent work seemingly failing to replicate the latter. Detailed analysis of methodological differences between studies reveals that the way experiments are set up explains whether sensorimotor cortex maps phonological information during speech perception or not. In particular, acoustic noise during the experiment and ‘motor noise’ caused by button press tasks work against the frontoparietal manifestation of phonemes. We highlight recent studies using sparse imaging and passive speech perception tasks along with multivariate pattern analysis (MVPA) and especially representational similarity analysis (RSA), which succeeded in separating acoustic-phonological from general-acoustic processes and in mapping specific phonological information on temporal and frontoparietal regions. The question about a causal role of sensorimotor cortex on speech perception and understanding is addressed by reviewing recent TMS studies. We conclude that frontoparietal cortices, including ventral motor and somatosensory areas, reflect phonological information during speech perception and exert a causal influence on language understanding.

## Introduction

Establishing links between the specifically human ability to speak and understand language and the underlying neuronal machinery of the human brain is a key to modern cognitive neuroscience. At the level of specific language sounds, or *phonemes*, such links were first suggested by magnetoencephalography (MEG) recordings which showed that neuromagnetic activity differed between vowel types (Diesch et al., 1996). This work was followed by demonstrations of distinct and phoneme-specific local activity patterns in the superior temporal cortex, close to auditory perceptual areas (Obleser et al., 2003, 2006; Obleser and Eisner, 2009). However, phonemes are abstract multimodal units interlinking what is heard with how to produce the acoustic signals, and even visual representations of the articulatory movement play a role in processing speech sounds (McGurk and MacDonald, 1976; Schwartz et al., 2004). Therefore, their neuronal correlates may not be locally represented in the brain in and close to the auditory-perceptual temporal cortex alone, but, instead, may be supported by distributed neuronal circuits that interlink acoustic perceptual and articulatory motor information (Pulvermüller, 1999; Pulvermüller and Fadiga, 2010; Schwartz et al., 2012; Strijkers and Costa, 2016).

**Figure 1:**
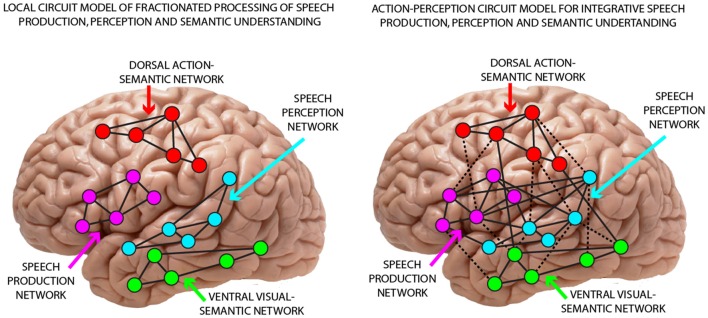
**Illustration of two competing theoretical positions regarding the role of temporal and frontal regions in speech perception. (Left)** The local fractionated circuit model implies segregated processes for speech production (in frontal and sensorimotor cortex) and speech perception (in superior temporal cortex). Accordingly, sensorimotor fronto-central speech production networks are not involved in and in particular, do not functionally contribute to phoneme processing. **(Right)** The action-perception-integration model postulates strong reciprocal links between superior-temporal speech perception and fronto-central production mechanisms yielding multimodal distributed neuronal circuits, which provide an interactive distributed neuronal basis for the production, perception and discrimination of phonemes.

That phonemic perceptual mechanisms link up with articulatory information processing in the mind and brain had long been stated by biological and cognitive models of speech processing. In particular Fry’s (1966) early model postulated sensorimotor articulatory-acoustic mechanisms and also the Motor Theory of Speech Perception (Liberman et al., 1967; Liberman and Whalen, 2000) linked phonemic production with perception, although other statements immanent to that theory—about the modularity of speech processing and the primacy of the speech motor module for perception—appear problematic today (Galantucci et al., 2006; Pulvermüller et al., 2006). Contrasting with the cross-modal links suggested by biological and motor theories, a classic position in the neuroscience of language had been that speech motor and speech perception networks are relatively independent from each other (Wernicke, 1874; Lichtheim, 1885), a position also inherited by more recent approaches. As one example, Hickok, (2014, p. 181). views the posterior superior temporal sulcus as the locus for phonemes and as “the real gateway to understanding”. Today, two diverging positions dominate discussions about the brain basis of phonemes (Figure 1). In one view, phonemic speech perception circuits are located in temporal and temporo-parietal cortex and are functionally separate from speech production circuits in inferior frontal and articulatory areas. We call this the “local fractionated circuit model” of speech perception and production, because, in this view, the temporal speech perception network would realize speech recognition on its own (local fractionation) and speech production circuits in fronto-parietal cortex (or “dorsal stream”) are considered to play “little role in perceptual recognition” (Hickok, 2014, p. 239)^1^. Speech production and perception are thus viewed as independent processes, mapped onto separate brain substrates with no significant interaction between them, hence the term “fractionated circuit model”. In contrast, the “action-perception integration model” postulates strong reciprocal links between speech perception and production mechanisms yielding multimodal distributed neuronal circuits, which provide the neuronal basis for the production, perception and discrimination of phonemes. These distributed multimodal circuits encompass acoustic perceptual mechanisms in temporal cortex along with articulatory sensorimotor information access in fronto-parietal areas^2^. Thus, in contrast to Liberman’s pure motor theory, which viewed articulatory gestures, i.e., motor units, as the central unit of speech perception, modern neurobiological theories of speech perception emphasize the interplay between perceptual and motor processes, positing that language processing relies on action-perception circuits distributed across auditory and motor systems (Pulvermüller and Fadiga, 2010, 2016).

From an integrative action-perception perspective, the fronto-parietal sensorimotor system appears well suited for processing fine-grained differences between speech sounds, because the muscles and motor movements relevant for the articulation of speech sounds have different and well-investigated cortical loci side by side (Penfield and Rasmussen, 1950; Bouchard et al., 2013). Neighboring body parts are controlled by adjacent locations of the motor and premotor cortex (PMC) and a similar somatotopic relationship holds in the somatosensory cortex, where the sensations in adjacent parts of the body are represented side-by-side. Different articulators such as the lips, jaw and tongue are localized from top to bottom in the so-called “motor strip”, thus predicting that a phoneme strongly involving the lips—such as the [+bilabial] phoneme /p/—is cortically underpinned by relatively more dorsal neuronal assemblies than a tongue related phonological element—such as the [+alveolar] phoneme /t/. Apart from predominant articulator involvement *per se* (e.g., tongue vs. lips), different actions performed with the same articulator muscles may have their specific articulatory-phonological mappings in the motor system (Kakei et al., 1999; Graziano et al., 2002; Pulvermüller, 2005; Graziano, 2016), thus possibly resulting, for example, in differential cortical motor correlates of different tongue-dominant consonants (/s/ vs. /∫/) or vowels (features [+front] vs. [+back] of /i/ vs. /u/). Crucially, in the undeprived language learning individual, (a) phoneme articulation yields immediate perception, so that articulatory motor activity is immediately followed by auditory feedback activity in auditory cortex, and (b) the relevant motor and auditory areas are strongly connected by way of adjacent inferior frontal and superior temporal areas, so that (c) well-established Hebbian learning implies that auditory-motor neurons activated together during phoneme production will be bound together into one distributed neuronal ensemble (Pulvermüller and Fadiga, 2010).

In this *action-perception integration perspective*, speech sounds with different places of articulation have their cortical correlates in different activation topographies across superior-temporal and fronto-parietal areas, including the articulatory sensorimotor cortex. If this statement is correct, it should be possible (i) to see motor activity during speech perception, phoneme recognition and language understanding^3^, and (ii) phonemes with different places of articulation and articulator involvement should differentially activate subsections of the articulatory motor system. Furthermore, distributed sensorimotor cortical circuits for phonemes imply (iii) that causal effects on speech perception and understanding can originate not only in auditory cortex and adjacent secondary and “higher” multimodal areas, but also in frontoparietal areas in and close to sensorimotor ones. As the motor and the somatosensory cortex have parallel somatotopies and with every articulator movement (performed under undeprived conditions) there is specific stimulation of the corresponding somatosensory cortex as well, this position predicts not only specific motor cortex activation in speech perception, but, in addition, somatosensory cortex activation. Indeed, there is evidence for a role of somatosensory systems both in speech production (Tremblay et al., 2003; Bouchard et al., 2013) and perception (Möttönen et al., 2005; Skipper et al., 2007; Ito et al., 2009; Nasir and Ostry, 2009; Correia et al., 2015; Bartoli et al., 2016). The motor and somatosensory system may already be important for speech perception early in life, since pacifiers blocking specific articulator movements were shown to affect the discrimination of speech sounds even in the first year (Yeung and Werker, 2013; for review see Guellaï et al., 2014).

To sum up, a major controversy between the competing models (Figure 1) surrounds the involvement of the sensorimotor cortex and adjacent areas in the fronto-parietal cortex (or “dorsal stream”) in speech perception and understanding. While both agree on a role of temporal areas in speech recognition, the “fractionated” model states independence of speech perception from fronto-parietal circuits, whereas the integrative action-perception perspective predicts interaction, and hence, additional involvement of fronto-parietal including sensorimotor cortices in speech perception and understanding. In this review article, we will evaluate the empirical results that speak to this controversy in an attempt to settle the debate.

## Auditory/Temporal and Sensorimotor/Fronto-Parietal Activation in Speech Perception

When speech sounds embedded in meaningless syllables are presented to the ears, functional magnetic resonance imaging (fMRI) reveals widespread activation in both temporal and frontal areas (for a meta-analysis, see Vigneau et al., 2006). Activation in the auditory cortex and surrounding areas of superior and middle temporal cortex is not surprising because most of the afferent ‘cables’ of the auditory pathway conveying sound information, from the ears terminate in superior temporal primary auditory cortex (Brodmann Area (BA) 41), from where activation spreads to adjacent and connected areas. Some of this activity, especially in the left language-dominant hemisphere, but also to a degree in the other one, is specific to speech, as is evident from comparisons of speech-sound elicited activity with that to noise patterns matched to speech (Scott et al., 2000; Uppenkamp et al., 2006). Some discrepancy still exists between data showing that speech specific activity is primarily present in anterior superior temporal cortex (Scott and Johnsrude, 2003; Rauschecker and Scott, 2009) or, alternatively, in posterior superior and middle temporal cortex (Shtyrov et al., 2000, 2005; Uppenkamp et al., 2006). Therefore, a role of both anterior and posterior temporal areas in processing speech sounds needs to be acknowledged (DeWitt and Rauschecker, 2012).

However, in addition to temporal areas, the frontal and sensorimotor cortex seems to equally be activated in speech processing. Early fMRI studies could already demonstrate general activation in the left inferior frontal cortex during passive speech perception (Poldrack et al., 1999; Benson et al., 2001). In a seminal study, Fadiga et al. (2002) applied magnetic stimulation to the articulatory motor cortex and showed that motor-evoked potentials (MEPs) in the tongue muscle are specifically enhanced when subjects listen to speech containing phonemes that strongly involve the tongue—in particular the rolling /r/ of Italian—and are enhanced even more speech sounds embedded into meaningful words (but see Roy et al., 2008). As this evoked-potential enhancement is likely due to increased activity in tongue-related motor and premotor cortex, it has been interpreted as a confirmation for motor system activation in speech perception. Further converging evidence came from studies using a range of methods, including fMRI and MEG/electroencephalography (EEG) with source localization (e.g., Watkins et al., 2003; Watkins and Paus, 2004; Wilson et al., 2004), and it could be demonstrated that activation spreads rapidly from the superior temporal to inferior frontal areas (Pulvermüller et al., 2003, 2005; see Tomasello et al., 2016, for converging evidence from computational modeling). Sound-evoked activity in the motor or sensorimotor system is not specific to speech sounds as compared with other acoustic stimuli, because similar patterns of motor activation have also been seen for nonlinguistic sounds, in particular for the sounds of mouth-produced or manual actions (Hauk et al., 2006; Scott et al., 2006; Etzel et al., 2008). However, apart from showing motor involvement in speech perception, Fadiga et al.’s (2002) work and related studies suggested specificity of activation at a more fine-grained level. In particular, the tongue-related articulatory-phonological nature of the /r/ sound may have contributed to localization specificity^4^. As we discuss below, this was investigated in detail in further studies.

## Does Sensorimotor Cortex Contain Phonological Information Relevant for Speech Perception?

**Figure 2:**
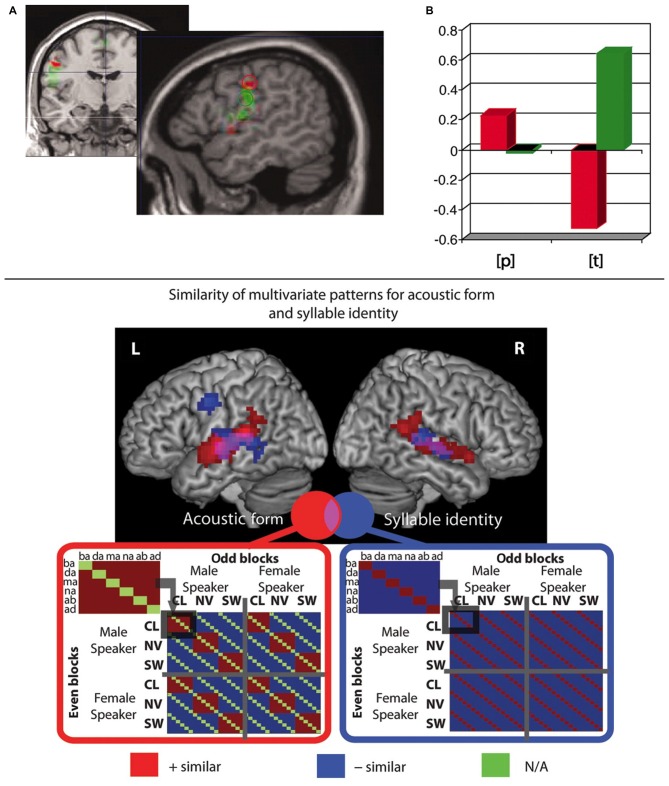
**Functional magnetic resonance imaging (fMRI) studies showing presence of phoneme-related information in motor systems during passive syllable perception. (Top) (A)** Regions of interest (ROIs) were derived from non-linguistic minimal lip and tongue movements. Lip ROI shown in red, tongue ROI in green. **(B)** Differential activation (arbitrary units) in those same ROIs during passive perception of lip- and tongue-related phonemes /p/ and /t/, indicating an interaction between ROI and place of articulation (PoA) of the perceived phoneme. Adapted from Pulvermüller et al. (2006; Figure 2), Copyright (2006) National Academy of Sciences, Washington, DC, USA. **(Bottom)** Representational similarity analysis (RSA) revealed that in pre- and postcentral motor regions the similarity of multivariate patterns reflects syllable identity, but not acoustic form; in contrast, in temporal regions, the similarity of patterns reflects both acoustic form and syllable identity. Patterns in precentral gyrus additionally reflect phoneme identity and CV structure (not shown in figure). Adapted from Evans and Davis (2015; Figure 3).

Some fMRI studies investigated whether, during speech perception, activity in frontoparietal and articulatory motor areas reflects phonological information, in particular about the phonemic features “place of articulation” (Pulvermüller et al., 2006; Raizada and Poldrack, 2007) and “voicing” (Myers et al., 2009). Pulvermüller et al. (2006) had subjects attentively listen to syllables starting with a lip-related bilabial /p/ or a tongue-related alveolar phoneme /t/. In the absence of any overt motor task, stimuli were passively presented during silent breaks where the MRI scanner was switched off, using a technique known as “sparse imaging” (Hall et al., 1999; Peelle et al., 2010), so as to allow speech perception without scanner noise overlay. After the linguistic perception part of the experiment, participants produced non-linguistic minimal lip and tongue movements and these movement localizer tasks were used to define lip and tongue regions of interest (ROIs), in sensorimotor cortex. When using these ROIs, and also when examining a range of subsections of the precentral cortex, the authors found that during perception of syllables starting with lip-related and tongue-related sounds, the corresponding relatively more dorsal vs. ventral sectors of sensorimotor cortex controlling those articulators were differentially activated. In other words, the motor cortex activation as a whole contained information about the place of articulation of the perceived phonemes (see Figure 2 top).

In recent years, the univariate fMRI studies of the brain correlates of speech perception were complemented by experiments using the novel analysis method of multivariate pattern analysis, or MVPA (Haxby et al., 2001; Norman et al., 2006; Haynes, 2015). This method offers a way of testing whether fine-grained voxel-by-voxel activation patterns within specific brain areas contain information about stimulus types, for example about phonetic and phonemic features of speech. Initially, the application of MVPA to fMRI activity in studies on phonological processing focused on temporal cortex, where successful decoding of vowel identity could be demonstrated (Formisano et al., 2008). Recently, this approach has been extended to activity not only in temporal, but also in fronto-parietal areas (Arsenault and Buchsbaum, 2015; Correia et al., 2015; Evans and Davis, 2015). Arsenault and Buchsbaum (2015) found a reliable place of articulation classification throughout superior and middle temporal cortex and in the left subcentral gyrus, an area at the intersection of precentral and postcentral cortices also active during articulation (Huang et al., 2002; Bouchard et al., 2013; Bouchard and Chang, 2014). However, these authors did not report reliable phonetic feature classification in the precentral motor cortex or inferior frontal cortex. Correia et al. (2015) trained classifiers on one phonetic feature using specific phonemes (e.g., place of articulation with stop consonants) and tested if performance, generalized to the same feature exhibited by different phoneme types (e.g., fricatives). Such cross-phoneme-type generalization was successful in a large sensorimotor region, including precentral motor regions, IFG, and the postcentral somatosensory cortex.

An innovative study by Evans and Davis (2015) used MVPA of phonological processing and employed representational similarity analysis (RSA; Kriegeskorte et al., 2008; Kriegeskorte and Kievit, 2013). This approach allows for testing models predicting the degree of similarity of neuronal patterns elicited by multiple pairs of stimuli. Using a “searchlight” approach, one can then calculate the “representational (dis)similarity” of neuronal patterns associated with different stimuli and see which of several models of predicted similarity most closely resembles the actual observed similarities of neuronal activity patterns. Evans and Davis (2015) tested models which predicted similarity according to acoustic features (e.g., noise or speaker identity) or phonemic properties (e.g., phoneme identity and place of articulation). By using these advanced image analysis methods (MVPA and RSA) the authors were able to disentangle brain activity patterns related to acoustic vs. phonemic similarity, an important issue previously not addressed by most previous imaging studies. Their results revealed that local neuronal activations reflect a graded hierarchy: in primary auditory cortex, neural patterns code for the acoustic form of speech only, irrespective of phonemic features. In bilateral superior temporal cortex, both acoustic and phonemic information is coded. Finally, in left precentral gyrus, the highest degree of abstraction is found, with patterns reflecting phonemic aspects exclusively (phoneme and syllable identity and consonant-vowel structure; see Figure 2 bottom). In sum, the majority of studies report phoneme mapping across a fronto-parieto-temporal perisylvian region and some innovative recent findings from RSA indicate that the motor system of the human brain is unique in mapping phonemic properties of speech relatively independent of acoustic features.

## Some Discrepancies Between Recent Findings

In a recent study, Arsenault and Buchsbaum (2016), tried to replicate Pulvermüller et al.’s (2006) univariate results on double dissociations between activation for tongue- and lip-related (alveolar/bilabial) speech sounds and conducted additional multivariate analyses. They report a failure to find such a double dissociation in the precentral gyrus, both when defining the ROIs based on coordinates taken from the original study and when using their own motor localizer ROIs. As true replication failures may be of significance, a second look at both studies is warranted. Closer inspection in fact shows major differences between the original and the attempted replication. Whereas Pulvermüller et al. (2006) had chosen a localizer task with only minimal articulator movements (to foster focal brain activation), such a task was not included in the new attempt. Rather, Arsenault and Buchsbaum (2016) based their own ROIs on a task requiring silent, but overt articulation of lip- and tongue-related phonemes (/p/ and /t/), a strategy which had not led to significant results in the earlier work. Secondly, Pulvermüller et al. (2006) used 5 and 8 mm ROIs, whereas Arsenault and Buchsbaum (2016) used 10 mm ROIs. Thirdly, whereas Pulvermüller et al. (2006) refrained from using an overt motor task in the speech perception condition—to avoid general task-related activation in the motor system—Arsenault and Buchsbaum’s (2016) subjects had to perform a button press on some trials. And finally, Pulvermüller et al. (2006) had spent effort to reduce scanner noise by applying sparse imaging techniques, and additionally used matched noise stimuli as a baseline for the speech perception condition, so as to allow for good signal-to-noise ratios in the speech-evoked hemodynamic response. In contrast, Arsenault and Buchsbaum (2016) presented their sounds during scanning so that all phoneme stimuli were overlaid by scanner noise. Considering these substantial differences between studies, the more recent work appears as a replication failure in two senses, with regard to the results and methods of the pre-existing work. Below, we present an analysis of the recent literature to find out which of the methodological aspects of Arsenault and Buchsbaum’s (2016) work might be responsible for the failure to replicate phoneme-related motor system activity (see the following section on “The Role of Scanner Noise”).

Apart from their purported replication attempt using univariate methods, Arsenault and Buchsbaum (2016) analyzed their data using MVPA. They trained a classifier on a subset of the perception trials and tested it on a different subset. Instead of a searchlight approach, they tested the classifier in three anatomically defined ROIs, in the precentral and central sulcus as well as in the postcentral somatosensory cortex. Although classification was unsuccessful in the precentral and central ROIs, results revealed significant decoding of place of articulation in left postcentral somatosensory cortex, in line with the findings by Correia et al. (2015). We also note that explaining the presence of articulator-related information in somatosensory cortex requires the invocation of motor mechanisms because the motor movements of the different articulators are causal for any specific somatosensory sensations related to speech sounds—hence the need for including somatosensory cortex in integrative action-perception models of language (see “Introduction” Section; Pulvermüller, 1992, 2013; Pulvermüller and Fadiga, 2010). In finding no MVPA mapping of phonological information in the motor system, Arsenault and Buchsbaum’s (2016) results are in apparent contrast with the work by Correia et al. (2015) and Evans and Davis (2015) discussed above. We now turn to possible explanations of the observed discrepancies.

## The Role of (Scanner) Noise and Overt Motor Tasks

In order to explain the discrepancies in results about the motor system’s role as an indicator of phoneme processing, it is necessary to pay special attention to subtle but possibly crucial differences between studies. In Table 1, we compiled a list of fMRI studies that found phonology-related information in specific cortical areas during (mostly passive) speech perception. The table lists studies that investigated the cortical loci of *general phoneme-related activity* during speech perception (studies 1–5) as well as activity carrying *specific phonological information* (studies 6–15), for example, activation differences between phonemes, phonological features and/or feature values (such as [+bilabial] or [+front]). Comparing studies against each other shows that the crucial methodological factors which predict acoustically induced phonological activation of, and information in, fronto-parietal areas are: (i) the use of “silent gap”, or “sparse” imaging (Hall et al., 1999; Peelle et al., 2010) and (ii) the absence of a requirement to perform button presses during the experiments. Both of these features are amongst those that distinguished Arsenault and Buchsbaum (2016) from Pulvermüller et al. (2006).

**Table 1:** **Overview of functional magnetic resonance imaging (fMRI) studies investigating involvement of inferior frontal, sensorimotor and inferior parietal systems in syllable perception**.

No.	Study	Stimuli features investigated	Phonetic	Task	Button presses	Sparse imaging	Analyses	Baseline	Activation/Decoding found in …		
									Prefrontal areas	Motor areas	Somatosensory and inferior parietal areas
1	Benson et al. (2001)	15 C/VC/CVC syllables	n/a	none	never	yes	univariate	non-speech tones	left BA 9, 10	left BA 6	left SMG (BA 40)
2	Wilson et al. (2004)	/pa/, /gi/	n/a	none	never	no	univariate	rest/silence	not reported	ventral (v) BA 4, 6	right SMG (BA 40)
3	Wilson and Iacoboni (2006)	50 consonants embedded between two /α/ vowels	n/a	none	never	no	univariate	rest/silence	not reported	v BA 4, 6	not reported
**4**	Szenkovits et al. (2012)	monosyllabic pseudowords	n/a	one-back repetition detection task	**10% of trials**	**no**	univariate	non-speech buzzes	not reported	not reported^d^	not reported
5	Grabski et al. (2013)	9 vowels	n/a	none	never	yes	univariate	rest/silence	BA 44, left BA 45	right BA 6	not reported
6	Pulvermüller et al. (2006)	/pæ/, /tæ/, /pI/, /tI/	Place	none	never	yes	univariate, ROI-based^a^	matched noise stimuli	not reported	left v BA 4, 6 (differential activation of lip vs. tongue regions)	not reported
7	Raizada and Poldrack (2007)	/ba/, /da/	Place	detect occasional quieter stimulus	6% of trials	yes	repetition adaptation	n/a	left middle frontal cortex (amplification of response to stimulus pairs differing in place)	not reported	left SMG (amplification of response to stimulus pairs differing in place)
8	Myers et al. (2009)	/da/, /ta/	Voicing	detect occasional high-pitched stimulus	37.5% of trials	yes	repetition adaptation	n/a	left inferior frontal sulcus (release from adaptation only for stimuli differing in voicing)	not reported	not reported
9	Lee et al. (2012)	10 CV syllables on /ba/-/da/ continuum	Place	detect occasional quieter stimulus	11% of trials	yes	MVPA (searchlight)	n/a	left BA 44 (decoding of place)	left pre-SMA (decoding of place)	not reported
10	Chevillet et al. (2013)^b^	/da/–/ga/ continuum	Place	dichotic listening (detect in which ear the sound persisted longer)	always	yes	repetition adaptation	n/a	not reported	left BA 6 (release from adaptation for stimulus pairs differing in place of articulation)	not reported
**11**	Du et al. (2014)	/ba/, /ma/, /da/, /ta/	Place	active syllable identification (4-AFC)	**always**	**no** (but scanner noise attenuation by 25 dB)	MVPA (searchlight)	n/a	Insula/Broca’s area (decoding of place)—at low/moderate noise levels only^e^	left v BA 6 (decoding of place)—at low noise levels only^f^	left inferior parietal lobule (decoding of place)—at low noise levels only
**12**	Arsenault and Buchsbaum (2015)	16 CV syllables	Place, manner, voicing	gender identification task	**always**	**no**	MVPA (ROI-based)	n/a	not reported	not reported	left subcentral gyrus (decoding of place)
13	Evans and Davis (2015)	/ba/, /da/, /ma/, /na/, /ab/, /ad/	Place, manner, phoneme identity, CV structure (CV vs. VC)	one-back repetition detection task	8% of trials	yes	MVPA-based RSA (searchlight)^c^	rest/silence	not reported	left precentral gyrus (decoding of syllable and phoneme identity and CV structure)	left postcentral gyrus (decoding of syllable identity)
14	Correia et al. (2015)	24 CV syllables	Place, manner, voicing	none	never	yes	MVPA (searchlight)	n/a	IFG (decoding of place/manner)	right inferior precentral gyrus (decoding of place)	postcentral gyrus, SMG (decoding of place/manner)
**15**	Arsenault and Buchsbaum (2016)	8 CV syllables: /ba/, /pa/, /va/, /fa/, /da/, /ta/, /za/, /sa/	Place (manner/voicing not analyzed)	detect occasional blank trials	**11% of trials**	**no**	univariate and MVPA (both ROI-based)	n/a	not reported	not reported	left postcentral gyrus (decoding of place)

**Figure 3:**
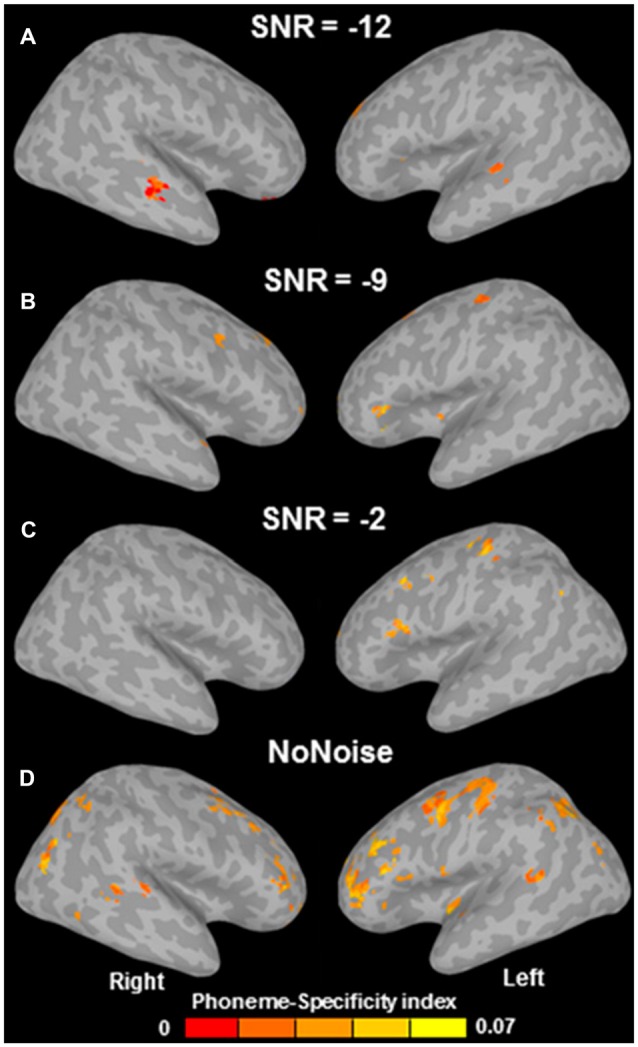
**Multivariate pattern analysis (MVPA) phoneme-specificity maps as a function of signal-to-noise ratio (SNR; in dB).** A more negative SNR indicates more additional noise on top of scanner noise attenuated by 25 dB (which was always present, even in the “no noise” condition). Successful MVPA decoding of phoneme identity in ventral premotor cortex (PMC) can only be seen in the “no noise” condition **(D)**, whereas with increasing noise **(A–C)**, decoding is unsuccessful in ventral PMC, but still successful in dorsal PMC and inferior frontal regions (see main text for detailed discussion). Adapted from Du et al. (2014; Figure S4).

*^a^Differential activation of subregions (lip vs. tongue) in left BA 4, 6 depending on place of articulation (lip vs. tongue; see Figure 2 Top). ^b^See also Alho et al. (2016) for similar results from MEG. ^c^Representational similarity analysis (RSA) used to disentangle acoustic and phonological similarity. ^d^Not reported in whole-brain analysis; but note that in a ROI-based analysis a correlation between working memory abilities and motor/premotor activation in speech perception was found. ^f^In dorsal BA 6 (PMC) decoding was still observed at higher noise levels (see Figure 3), but this might reflect classification of responses rather than speech perception (see main text for discussion). ^e^Due to the active identification task on every trial, this might reflect decision-related processes or response selection/preparation (see main text for discussion)*.

### The Role of Scanner Noise

Why would avoiding scanner noise be so important for finding brain activation related to speech perception in frontal areas? Arsenault and Buchsbaum (2016) argue that “according to previous literature, the background scanner noise […] should actually have *increased* the role of the PMC in speech perception”. However, a closer look at the literature shows that the reverse likely applies; Table 1 shows that those studies which avoided scanner noise, button presses, or both (No. 1–3, 5–10, 13–14) all found activation (or MVPA decoding) in left motor cortex or IFG during speech perception; in contrast, those studies where both scanner noise and button presses were present (No. 4, 11, 12, 15, marked bold) found no involvement of left frontal or motor regions. The only exception to this rule is study 11 (Du et al., 2014), which reports precentral phonemic information in spite of noise and button presses on every trial. Crucially, however, and in contrast to Arsenault and Buchsbaum’s (2016) statement, Du et al. (2014) found phoneme-related information in the ventral PMC (vPMC) only at the lowest noise level (headphone-attenuated scanner noise with no additional noise; Figure 3D); at higher noise levels, successful phoneme classification could not be shown in vPMC anymore (Figures 3A–C), but still in dorsal PMC (dPMC). They conclude that “adding noise weakened the power of phoneme discrimination in almost all of the above mentioned areas [see Figure 3D] except the left dorsal M1/PMC which may index noise-irrelevant classification of button presses via the right four fingers” (Du et al., 2014; p. 7128). This caveat is likely given that there was a one-to-one-mapping between response buttons and phoneme category and this wasn’t counterbalanced in Du et al.’s study. Decoding in inferior frontal areas (insula/Broca’s region) was somewhat more robust to noise. However, in contrast to all other studies in Table 1, Du et al. (2014) used an active syllable identification task on every trial; it is therefore unclear whether decoding in these areas reflects phonological information or, alternatively, decision-related processes or response selection/preparation (see Binder et al., 2004). In contrast, of particular interest for articulatory information are precentral motor areas—those which were the focus of Arsenault and Buchsbaum’s (2016) investigation; crucially, in these areas (as well as in superior temporal and inferior parietal regions), Du et al. (2014) found decoding to be most fragile, appearing only at the lowest noise levels.

Therefore, taking into consideration the caveats about Du et al.’s (2014) design, the following tentative conclusions can be offered: speech motor systems, but equally inferior parietal areas and superior temporal cortex—the latter being a site widely agreed to contribute to phonological processing—index phonological information processing only if the speech is presented without noise or with only moderate noise overlay.

Still, some studies reported that a contribution of frontal or motor systems *further increases* when stimuli become moderately more difficult to understand, for example, because of noise overlay (Murakami et al., 2011; Osnes et al., 2011; Adank et al., 2012; Hervais-Adelman et al., 2012), motor-perturbed speech distortions (Nuttall et al., 2016), increased subjective dissimilarity between the perceived and the listener’s own voice (Bartoli et al., 2015), or age-related hearing impairment (Du et al., 2016). However, an increasing contribution of motor systems with increasingly challenging listening conditions does not logically entail that this contribution is generally absent in non-noisy listening conditions^5^. This is seemingly at odds with some TMS studies that found no evidence for motor involvement in speech perception without noise (e.g., D’Ausilio et al., 2012). However, null effects in the absence of noise are equally open to an explanation in terms of ceiling effects (see also Sato et al., 2009, for a ceiling effect due to low task complexity). Note that normal speech is characterized by lots of redundancies due to co-articulation, requiring much information to be omitted before a measurable change in perception or comprehension performance can be found. Hence, if TMS to motor systems produces no effect in a task with high overall accuracy, this is likely a result of a ceiling effect or methodological factors (as, in general, TMS tends to produce weak effects) rather than indicating that motor systems’ contributions are indeed absent—apart from the obvious fact that absence of evidence in favor of an effect is no evidence of its absence. Taking a broader perspective, there is abundant evidence for motor systems activation during language processing in sparse-imaging fMRI experiments (see Table 1), as well as in other noise-free (and passive) tasks (Fadiga et al., 2002; Möttönen et al., 2013, 2014; Shtyrov et al., 2014; Grisoni et al., 2016).

In summary, motor systems’ contributions to speech processing tend to show up already with no noise and might further increase with moderate noise overlay. However, with too strong noise overlay (which non-attenuated scanner noise might constitute), this contribution disappears again. This observation is problematic for models viewing perceptually-induced motor system activation as correlate of a prediction process *only* effective under noisy or otherwise challenging perceptual conditions (Hickok, 2015)^6^.

### The Role of Overt Motor Tasks

We now turn to the second important methodological point, the role of overt motor responses (e.g., occasional or constant button presses). Arsenault and Buchsbaum’s (2016) study is subject to such a motor response confound. Subjects had to press a button occasionally on catch trials (11% of trials), to ensure they were paying attention. Therefore, subjects had to be prepared throughout the experiment to respond, thus leading to constant preparatory activity in the motor system. Such preparatory activity does not only involve the primary and pre- motor cortex, but, in addition, adjacent supplementary motor and prefrontal cortices as well, as is evident from studies investigating the so-called readiness potential and related preparatory brain indicators (Kornhuber and Deecke, 1965; Babiloni et al., 2001). Note that hand representations in somatosensory and motor areas lie side by side with articulator representations, especially of the lips. Presumably, preparatory neuronal activity in motor regions induced by a button press task causes a ceiling effect, which leads to a reduced chance of seeing small speech-sound induced articulator-related activity in motor cortex. Indeed, previous studies using lexical decision tasks requiring a button press also often found no evidence of semantically related activation in motor cortex, whereas most studies using passive paradigms found such “semantic somatotopy” (Carota et al., 2012; Kemmerer, 2015). This discrepancy is best explained by preparatory hand-motor activity (for discussion, see Pulvermüller et al., 2001). A similar effect could be at work both in Arsenault and Buchsbaum (2016) and in their earlier study (2015) which even required a button press on every trial (plus a gender identification task). This pattern of results is consistent with the statement that preparatory motor activity and hence overt button press tasks work against the detection of phonological information processing in the articulatory motor cortex. This position would also offer an explanation why Arsenault and Buchsbaum (2015, 2016), despite finding no MVPA decoding in precentral cortex, nonetheless reported successful discrimination in the postcentral somatosensory regions, where any preparatory motor activity is minimal or absent, hence not leading to a ceiling effect as in precentral motor regions.

In sum, a review of a range of neuroimaging experiments on speech processing shows that the factors noise overlay and overt motor tasks explain why some previous univariate and multivariate fMRI studies found evidence for phoneme-specific activation in frontal cortex, including Broca’s and precentral areas, and why others did not^7^. The mechanisms underlying these effects need further clarification, but a tentative mechanistic explanation can be offered in terms of acoustic phonemic signal-to-noise ratios reflected in the fronto-central cortex, which must decrease both with overlay of acoustic noise and ‘motor noise’ which may result from preparatory motor movements. These two factors, especially in combination (see studies 4, 11, 12, 15 in Table 1), seem to cause a loss of phoneme-related activation in frontal areas, which also explains the unsuccessful replication attempt of Arsenault and Buchsbaum (2016) and the discrepancies of their work with other recent studies (Correia et al., 2015; Evans and Davis, 2015)^8^. Therefore, a clear take home message from this review can be phrased as follows: in order to map the full cortical signature, including motor activity, of speech recognition and processing, it is advantageous to avoid (i) acoustic noise and (ii) overt motor responses. A further suggestion is to avoid tasks focusing attention on stimulus aspects which are not in focus (e.g., speaker identity when investigating phonological features), as this also has an impact on MVPA decoding (Bonte et al., 2014). An analogous suggestion may apply to other perception-related brain activity patterns as well.

## Excursus: Cross-Decoding from Miming to Perception as the Critical Test?

A methodologically innovative aspect of Arsenault and Buchsbaum’s (2016) study, compared to previous MVPA studies on this topic, was that they also used multivariate cross-classification, or cross-decoding (see Kaplan et al., 2015 for review). In this approach, a machine learning classifier is trained to distinguish a difference between types of stimuli in one condition or brain area and its performance is then tested on a different condition or brain area. Arsenault and Buchsbaum (2016) applied this logic to the difference between silent syllable articulation (“miming”) and speech perception conditions. Classifiers were trained on the distinction between bilabial and alveolar place of articulation (PoA) on the miming data; they then investigated whether that same classifier could decode PoA from the fMRI patterns in the speech perception condition as well. Crucially, this cross-modality decoding from miming to perception did not succeed, which, according to Arsenault and Buchsbaum (2016) would be “the critical test of motor theories of speech perception”.

This latter statement is problematic, however; no explanation is given as to why this cross-decoding should constitute “the critical test”. This view seems to imply that substantial similarities should exist between the cortical activity patterns seen during speech production and perception. In contrast, the crucial prediction of action-perception integration models of speech which was vindicated by Pulvermüller et al. (2006), was that phoneme perception involves access to multimodal phoneme representations which, due to their multimodal character, *include* neurons with articulatory function in the speech motor system (cf. Galantucci et al., 2006). The key finding (see Figure 2 top) was that lip and tongue regions of motor cortex were differentially activated during speech perception, indicating that “information about articulatory features of speech sounds is accessed in speech perception” (Pulvermüller et al., 2006, p. 7868). The link between perception and articulator movement conditions in Pulvermüller et al. (2006) consists in the fact that subregions of motor cortex (lip vs. tongue) were defined as ROIs based on the articulator movement localizer, and in the perception condition, these same ROIs exhibited similar differential activity depending on the perceived phoneme. Thus, what the conditions had in common was that both of them produced articulator-specific activation of subregions of motor cortex. But this does not suggest that there should be more general and wide-ranging similarities in neural activation patterns between these conditions. In fact, empirical evidence clearly shows large differences between speech production and perception. For example, the strong motor activity controlling overt articulator muscle movements during speech production is different from the slightly enhanced excitability of articulatory motor regions in speech perception (Fadiga et al., 2002) and clear dissociations at the level of neural activity have also been demonstrated using fMRI (Figure 1 in Pulvermüller et al., 2006; see also Markiewicz and Bohland, 2016). Apart from differences in *degree* of activation (e.g., motor activity being strong in production, but weak/sub-threshold in perception), further important differences between production and perception are obvious. For example, trivially, subjects are overtly moving their articulators in production, thus generating somatosensory self-stimulation, whereas both of these processes are absent in passive speech perception. Likewise, acoustic stimulation with speech sounds leads to acoustic processes not present during speech motor programming or silent articulation. Already due to these obvious cognitive-behavioral and related neurophysiological differences alone, significantly different neuronal activation patterns are to be expected between production and perception. However, such necessary differences cannot argue against shared auditory and sensory *mechanisms*, i.e., production and perception mechanisms may both involve the activation of shared action-perception circuits as one of their components.

In summary, it appears unreasonable to expect *identical* neural activation for motor action and concordant perception (in this case silent articulation or “miming” of speech sounds and their perception). Rather, the aspects of neural activity shared between perception and production can only be a subset of the total activity patterns present during both. Hence, when testing a classifier in a condition which shares only some of the relevant processes with the condition it was trained on, it is no surprise that cross-decoding is difficult. Such a result fits well with general observations from other MVPA studies, which found, firstly, that in general cross-decoding performance is reduced when performed across different modalities (auditory vs. written word presentation; Akama et al., 2012), but, critically, that cross-modal classification accuracies are often asymmetrical depending on cognitive features. For example, Cichy et al. (2012) found that cross-decoding from imagery to perception was less successful than vice versa, supposedly because the neural patterns of imagery are only a subset of those of perception (see also de Borst and de Gelder, 2016). Similarly, Oosterhof et al. (2012) found that cross-decoding was more successful when training on imagery and testing on action execution than vice versa. Hence, it appears as generally difficult to succeed with cross-decoding of perceptual/cognitive patterns from motor tasks; in the case of Arsenault and Buchsbaum (2016) additional complications were introduced because a motor response task was present in the perception condition, but not the miming condition; conversely, head motion induced artifacts might have been present in the miming but not the perception condition. Hence, further differences between the two conditions were introduced, which could contribute to the classifier learning features which are discriminative only in miming but not in perception and vice versa. Therefore, both the motor response task, while being problematic in itself (as discussed above), and the fact that overt articulation rather than minimal articulator movements were used, likely contributed to difficulties in multivariate cross-decoding by adding further differences between conditions. In conclusion, Arsenault and Buchsbaum’s (2016) lack of success in decoding speech perception information based on miming data does not come as a surprise and cannot be interpreted as evidence for or against specific neurocognitive models.

## The Functional Relevance of (Phonological Information In) Sensorimotor Cortex for Speech Perception and Understanding

The neurophysiological experiments reviewed above show that phonological information about perceived speech, including abstract phonemic distinctive features such as place of articulation, is reflected in differential patterns of activation in motor cortex. These results are of great theoretical interest, as they help to decide between competing theories that view speech perception either as a fractionated sensory process or as an interactive mechanism involving both action and perception information and mechanisms.

However, the mere activation of sensorimotor cortex in perception could be due to intentional articulatory activity, which adds to the perception mechanism from which it is otherwise functionally divorced. Such motor activity may be sub-threshold and may thus appear while no corresponding movement or muscle activity occurs. Motor activity during, but entirely independent of perception, may be linked to motor preparation or to predicting future perceptual input. To judge this possibility, it is critical to find out whether perceptually-induced motor activation indeed carries a more general function in speech processing. Already some brain activation studies suggest a functional role of motor cortex activation in speech processing. One study found that the magnitude of speech-evoked motor activity reflects working memory capacities of experiment participants (Szenkovits et al., 2012). Other work showed that perceptually-induced motor activation reflected the type of language learning by which novel “pseudo-words” had been acquired. Fronto-central cortical responses to novel sequences of spoken syllables increased when subjects familiarized themselves with these items by repeated articulation, whereas the passive perceptual learning of the same speech items did not lead to comparable sensorimotor activation (Pulvermüller et al., 2012; Adank et al., 2013). Further indication of functional contributions of motor systems to speech perception and comprehension comes from the observation that practice in producing unfamiliar sounds or accents significantly improves their discrimination/comprehension (Catford and Pisoni, 1970; Adank et al., 2010; Kartushina et al., 2015). Similarly, learning-induced plasticity in the motor system has been shown to alter speech percepts (Lametti et al., 2014). Therefore, perceptually induced motor activity may signify articulatory learning, working memory and long-term memory for speech sounds and spoken word forms^9^.

The strongest statement of an integrative active perception account, however, addresses a putative causal role of motor systems in the perceptual processing. Is the motor system causal for speech perception and understanding? To decide this crucial issue, a neuropsychological research strategy is required, which investigates whether functional changes in the sensorimotor cortex impact on speech perception. Indeed, TMS studies have demonstrated that the motor system has a causal influence on the discrimination and classification of speech sounds (Meister et al., 2007; D’Ausilio et al., 2009; Möttönen and Watkins, 2009; Krieger-Redwood et al., 2013; Rogers et al., 2014). Similar TMS modulation in phonological tasks has also been demonstrated for the inferior frontal and supramarginal gyrus (SMG; Hartwigsen et al., 2010a,b, 2016). Over and above any general causal influence on speech discrimination performance, a phoneme specific effect of local sensorimotor stimulation has been demonstrated by a number of TMS studies comparing speech sounds with different place of articulation (usually bilabials vs. alveolars, see D’Ausilio et al., 2009; Möttönen and Watkins, 2009). These studies showed a facilitation of phonological discrimination of “body-part congruent” sensorimotor stimulation on the processing of phonemes. For example, tongue area TMS specifically accelerated (and improved) the perceptual classification of “tongue sounds” such as /d/ and /t/. These results converge with the earlier fMRI study on the topographical specificity of the place of articulation of speech sounds in sensorimotor cortex. In addition to showing phoneme-specific topographic activation, they also indicate a causal role of motor cortex in perception.

As mentioned before, research addressing the causality question requires a neuropsychological research strategy whereby the manipulated independent variable is the change of brain states (e.g., by TMS) and the measured dependent variable is a behavioral response, for example the accuracy and/or latency of a button press. Therefore, all neuropsychological studies require an overt motor task and any task administered in an experimental laboratory is to a degree “unnatural”, such studies are open to criticisms. Researchers holding a critical attitude towards action-perception theory, for example Hickok (2014), choose to criticize the use of phoneme identification and discrimination tasks as “unnatural” and possibly engaging processes not required in everyday language use and understanding. This position does not come without any reason, as pressing a button labeled with the letter “p” or “d” is certainly not an activity normal listeners would frequently engage in when hearing and processing speech. In this context, it has been argued that TMS might not modulate perception, but rather decision-related processes instead. Different mappings on motor system areas might therefore reflect aspects of decisions, not phonological information. However, an explicit investigation of this issue using signal detection theory found that after TMS to lip motor cortex, changes in speech perception tasks are driven by changes in perceptual sensitivity, but not by decision-related processes such as response bias (Smalle et al., 2015). Furthermore, even in the absence of any task, Möttönen et al. (2013) found that an attention-independent neurophysiological index of speech sound processing known as the mismatch negativity or MMN (Näätänen et al., 1997), was reduced following TMS to lip motor cortex. This result shows that sensorimotor cortex stimulation modulates a major physiological marker of speech perception, even in the absence of a task, although a follow-up MEG study found that this modulation appeared relatively late and was not specific to the place of articulation (Möttönen et al., 2014). In sum, MMN studies indicate that articulatory motor cortex reflects speech sound processing, rather than decision related processes such as response bias, and that functional changes in this part of the motor system reduces neurophysiological correlates of speech sound processing.

**Figure 4:**
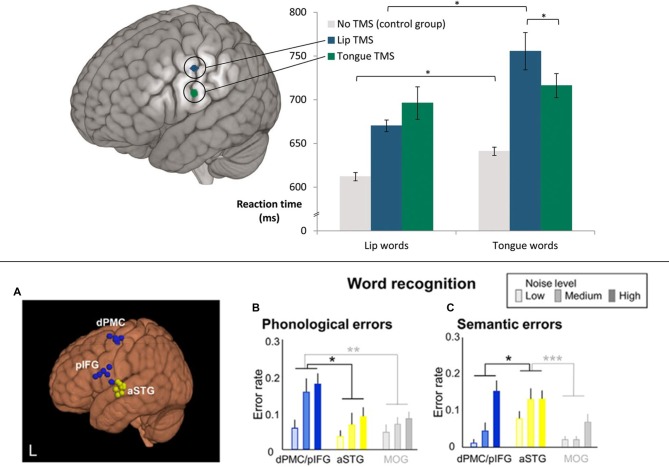
**Transcranial magnetic stimulation (TMS) studies showing causal effects of frontal cortex stimulation on speech comprehension (word-to-picture matching). (Top)** Double TMS pulses to different articulator representations in motor cortex (lip vs. tongue) led to relative facilitation in word comprehension responses for words starting with a phoneme related to the congruent articulator, as revealed by a significant interaction of stimulation locus and word type (“lip words” vs. “tongue words”). *p < 0.05. Adapted from Schomers et al. (2015; Figure 1) by permission of Oxford Univ. Press, material published under a CC-BY-NC license. **(Bottom)** A simultaneous virtual lesion in both dPMC and pIFG (using “double-knockout” thetaburst TMS) led to significantly increased semantic and phonological errors in word recognition (word-to-picture matching). *p < 0.05, **p < 0.01, ***p < 0.001. Adapted from Murakami et al. (2015; Figure 6).

One may still ask, however, how this TMS functional change relates to language comprehension under normal conditions, as speech sound discrimination tasks do not provide conclusive evidence about any causal role in language comprehension. The standard task with which psycholinguists investigate single word comprehension uses pictures and has subjects, select a picture related to a spoken word. This *word-to-picture-matching task* (WPMT) was applied recently in two TMS experiments. In one experiment (Schomers et al., 2015), pictures were shown whose typical verbal labels were phonological “minimal pairs” only differing in their word-initial phoneme, which was either a [+bilabial] lip-related or [+alveolar] tongue-related speech sound (for example, pictures of a deer and a beer were shown while the spoken word “deer” was presented). TMS to lip- and tongue-controlling precentral sulcus differentially influenced reaction times in the comprehension of spoken words starting with [+bilabial] and [+alveolar] phonemes, respectively (see Figure 4 top), thus demonstrating a causal role of sensorimotor cortex on speech comprehension. As in previous studies using sub-threshold single or double TMS, a relative facilitation effect was revealed by response times. In another recent experiment, Murakami et al. (2015) used a “double-knockout” thetaburst TMS protocol, a novel technique where two different brain areas are stimulated with bursts of theta frequency TMS pulses (Huang et al., 2005), causing long-lasting (up to 60 min) functional degradation simultaneously in both areas. After such “double-knockout” of both pIFG and dPMC an increase in phonological errors in a WPMT was observed (see Figure 4 bottom). Interestingly, this effect did not significantly interact with noise level, indicating that noise overlay was not a crucial factor in observing involvement of frontal areas in speech comprehension (see section on “The Role of Scanner Noise”).

In conclusion, sensorimotor articulatory cortex does not only reveal phoneme-specific activation signatures during speech perception, it also takes a differential phoneme-specific causal role in speech perception and word comprehension. Importantly, as both facilitation and error-induction could be observed in speech comprehension tasks, the causal role of sensorimotor cortex in perceptual tasks receives strong support.

## Conclusion

So, is the sensorimotor system relevant for speech perception and comprehension? Considering the evidence available across methods, studies and laboratories, this question receives a clear “Yes”. Still, noise overlay and motor tasks during speech perception may cancel any measurable phonologically related activation in the motor system, including multivoxel pattern information reflecting phonological specificity.

Evidence from univariate analyses of fMRI data has long shown that various parts of the speech motor system are activated during passive speech perception. Some of these studies even found specific phonological information, e.g., about place of articulation or voicing, present in these areas. Recently, several fMRI studies using MVPA replicated and extended the earlier findings. An open question that remains is what the precise role of the different regions in the sensorimotor system is, in particular the IFG, the premotor, primary motor and somatosensory cortices (see Hertrich et al., 2016, for a recent review on the role of the supplementary motor area). Mechanistic neurobiological models suggest that the roles of neurons in primary, secondary and higher multimodal areas in both frontal and temporal lobes can be understood in terms of distributed functional circuits within which distributional different patterns of activation are the basis of the perception, recognition and working-memory storage of phonemes and meaningful units (Pulvermüller and Garagnani, 2014; Grisoni et al., 2016).

Still, there is substantial divergence between some of the reported findings regarding the precise locations where phonological information can be detected in the neurometabolic response (see Table 1). We argue here that at least a significant portion of this variance can be explained by differences in methods, in particular by the features of scanner noise and preparatory motor activity. Activity in motor cortex, especially precentral gyrus, seems to be vulnerable to both (whereas activity close to auditory areas and in somatosensory cortex is not as much influenced by preparatory motor activity). Hence, in order to observe motor system activity in perception experiments, it is of the essence to reduce acoustic noise and ‘motor noise’ as much as possible, i.e., to use sparse imaging and avoid having subjects engage in (even only occasional) button presses throughout the experiment. Finally and most importantly, any discrepancies in fMRI results are secondary in light of clear evidence from TMS that modulation of sensorimotor and frontoparietal areas causes functional changes in speech perception and comprehension, both measured neurophysiologically (Möttönen et al., 2013, 2014) and behaviorally (D’Ausilio et al., 2009; Möttönen and Watkins, 2009; Hartwigsen et al., 2010a,b, 2016; Rogers et al., 2014; Bartoli et al., 2015; Murakami et al., 2015; Schomers et al., 2015; Smalle et al., 2015).

## Author Contributions

MRS and FP analyzed and reviewed the relevant literature and wrote the article.

## Funding

Funding was provided by the Deutsche Forschungsgemeinschaft (DFG, Pu 97/16-1), the Berlin School of Mind and Brain, and the Freie Universität Berlin.

## Conflict of Interest Statement

The authors declare that the research was conducted in the absence of any commercial or financial relationships that could be construed as a potential conflict of interest.

## Footnotes

^1^But note that some other publications of the same author (Hickok et al., 2011; Hickok, 2015), while still proposing a fractionated model overall, do acknowledge that the speech motor system could exert a *modulatory* influence on speech perception by way of forward predictions, at least under certain specific task or contextual constraints.

^2^Such an action-perception integration perspective is not restricted to the speech domain, but equally applies to written word processing, where it has been demonstrated that reading letters activates the hand motor areas involved in writing (Longcamp et al., 2003).

^3^Note that obviously some suppression mechanisms are necessary to prevent overt motor movements/articulation during perception. In concrete implementations of action-perception integration models of language, the obvious differences between speech production and recognition (overt motor movements vs. open auditory ‘gates’) are implemented in terms of area-specific cortical regulation processes (Garagnani et al., 2008; Pulvermüller et al., 2014).

^4^Apart from phonological features, the high arousal and general motor activity associated with the rolling /r/ of Italian may be relevant for the observed specificity.

^5^Furthermore, it is well-known that completely noise-free and ‘perfect’ listening conditions rarely occur outside the context of laboratory experiments (D’Ausilio et al., 2012).

^6^Note that there are also similar proposals for motor-induced forward predictions in speech perception without constraints (Skipper et al., 2005, 2007), which sit well with the available data.

^7^A recent ECoG study (Cheung et al., 2016) reported superior-temporal along with pre- and post-central activation to single syllables presented in a button press task. Differences in brain responses were found reflecting the massive acoustic differences between stop and fricative sounds, but not for the fine acoustic differences between stop consonants with different place of articulation. As a button press task was used and data analysis focused on one specific neurophysiological measure, high frequency responses, these results do not motivate strong conclusions on motor recruitment in speech perception *per se* or the absence of phonemic discrimination in fronto-parietal cortex.

^8^Our literature review does not rule out other factors as additional explanatory variables, such as ROI-based vs. searchlight analyses, or searchlight size; for example, Correia et al. (2015) found successful classifier performance in left IFG only with a searchlight radius of 20 mm, but not 10 mm (see also Lee et al., 2012, for discussion of the influence of searchlight size on MVPA analyses).

^9^Park et al. (2016) suggest a functional role of the articulatory motor cortex even for audiovisual speech perception, since visually perceived lip movements were found to entrain oscillations in the lip motor cortex and the degree of coherence correlated with comprehension accuracy.

## Abbreviations

ECoG, electrocorticography; EEG, electroencephalography; MEG, magnetoencephalography; MEP, motor-evoked potential; MMN, mismatch negativity; fMRI, functional magnetic resonance imaging; MVPA, multivariate pattern analysis; ROI, region of interest; RSA, representational similarity analysis; TMS, rTMS, (repetitive) transcranial magnetic stimulation; (d/v)PMC, (dorsal/ventral) premotor cortex; (p)IFG, (posterior) inferior frontal gyrus; STG/S, superior temporal gyrus/sulcus; SMG, supramarginal gyrus; SMA, supplementary motor area; PoA, place of articulation.

## Abbreviations

ECoG: electrocorticography
EEG: electroencephalography
MEG: magnetoencephalography
MEP: motor-evoked potential
MMN: mismatch negativity
fMRI: functional magnetic resonance imaging
MVPA: multivariate pattern analysis
ROI: region of interest
RSA: representational similarity analysis
TMS, rTMS: (repetitive) transcranial magnetic stimulation
(d/v)PMC: (dorsal/ventral) premotor cortex
(p)IFG: (posterior) inferior frontal gyrus
STG/S: superior temporal gyrus/sulcus
SMG: supramarginal gyrus
SMA: supplementary motor area
PoA: place of articulation.
